# PLA2R‐Positive Membranous Nephropathy and AA Amyloidosis in an Ethiopian Patient With Chronic Hepatitis B: A Case Report

**DOI:** 10.1002/ccr3.72609

**Published:** 2026-04-24

**Authors:** Betelhem Abreham, Leja Hamza, Azeb Kebede, Daniel Rebuma Bekele, Zekarias Amdemariam

**Affiliations:** ^1^ St Paul Millennium Medical College Addis Ababa Ethiopia; ^2^ Zewditu Memorial Hospital Addis Ababa Ethiopia

**Keywords:** AA amyloidosis, case report, Ethiopia, hepatitis B virus, membranous nephropathy, nephrotic syndrome, PLA2R

## Abstract

The coexistence of primary phospholipase A2 receptor positive membranous nephropathy and AA amyloidosis in a patient with chronic hepatitis B is an exceedingly rare triad presenting a profound diagnostic and therapeutic challenge. A 38‐year‐old Ethiopian man with nephrotic syndrome and chronic hepatitis B had dual pathology on renal biopsy. Despite concurrent hepatitis B, C1q negativity and IgG4/PLA2R dominance favored a primary autoimmune process over viral‐associated secondary glomerulonephritis. Immunosuppressive therapy (rituximab then cyclophosphamide) achieved complete immunological remission with loss of glomerular PLA2R antigen, yet the patient had persistent heavy proteinuria. Repeat renal biopsy determined that residual proteinuria reflected irreversible structural damage rather than active autoimmune injury. This damage included increased segmentally sclerosed glomeruli (from 11% to 33.3%) with permanent disruption of the filtration barrier from residual amyloid deposits and fibrosis. In multiple pathology settings, outcomes depend on the independent progression of each disease process. Thus, an integrated approach combining longitudinal serology and histological follow‐up is essential for managing such complex cases.

## Key Clinical Message

Coexisting PLA2R positive membranous nephropathy and AA amyloidosis with chronic hepatitis B requires distinguishing viral associated disease from primary autoimmune pathology. Lack of proteinuria response to antivirals, IgG4/PLA2R immunopathology, and response to immunosuppression confirmed primary MN. Although immunosuppression achieved complete immunological remission and loss of glomerular PLA2R antigen, heavy proteinuria persisted due to irreversible structural damage. Repeat biopsy proved this proteinuria stemmed from residual amyloid deposits and increased global sclerosis rather than ongoing immunologic injury.

**Key Clinical Points:**

- In regions where HBV is endemic, chronic HBV infection may co‐exist with primary PLA2R‐positive MN rather than invariably causing secondary membranous nephropathy. The absence of C1q deposition and the dominance of the IgG4/PLA2R axis favor a primary autoimmune process. Furthermore, the absence of a clinical response to antiviral therapy, despite viral suppression, argues against secondary HBV‐associated membranous nephropathy and supports coincidental primary membranous nephropathy requiring immunomodulatory treatment.
- The co‐existence of AA amyloidosis in a patient with chronic HBV infection, in the absence of any other chronic inflammatory condition, implicates HBV as the underlying driver. Antiviral therapy to suppress HBV replication is the primary intervention, and a reduction in amyloid burden on repeat biopsy confirms treatment efficacy.
- Persistent proteinuria after treatment does not always signal treatment failure or active disease. Repeat renal biopsy in this case revealed that while the PLA2R antigen cleared (immunological remission), global glomerulosclerosis tripled and amyloid deposits persisted. This distinguishes irreversible structural damage from active immunologic injury, preventing the risk of unnecessary and potentially harmful over‐immunosuppression.
- In patients with dual renal pathologies (membranous nephropathy and amyloidosis), the dominant disease driving proteinuria cannot be determined by clinical parameters alone; histologic follow‐up is mandatory. An integrated approach combining longitudinal serological monitoring, IgG subclass analysis, and histological follow‐up is critical for the accurate management of overlapping autoimmune and infectious renal diseases.

## 1 Introduction

Membranous nephropathy (MN) is an immune‐mediated glomerular disease characterized by subepithelial deposits of immunoglobulin G (IgG) and complement components between the glomerular basement membrane (GBM) and podocytes, leading to proteinuria and a variable risk of progression to end‐stage renal disease [1, 2, 3]. Histopathologically, it is defined by thickened glomerular basement membranes with these immune complex deposits, which are detectable by immunofluorescence and electron microscopy [4]. It can be primary or secondary to infections, systemic autoimmune diseases, drugs, malignancy, or dietary antigens [1, 5, 6]. Primary MN, accounting for approximately 70%–80% of cases, is strongly associated with autoantibodies against the phospholipase A2 receptor (PLA2R) on podocytes [7]. The presence of PLA2R is a hallmark of primary MN; however, its occurrence in secondary forms, particularly those associated with chronic infections like hepatitis B virus (HBV), suggests a complex interplay of immune dysregulation where the infection may trigger an autoimmune response [8]. In fact, studies have demonstrated that PLA2R can co‐localize with HBV antigens in glomerular deposits, and a significant proportion of patients with HBV‐MN have both subepithelial PLA2R antigen and circulating anti‐PLA2R antibodies, indicating HBV may be a trigger for this autoimmunity [8]. Some studies identified PLA2R antibodies in 20% of patients with secondary membranous nephropathy like hepatitis B [9].

Renal disease may occur in 3%–5% of patients with chronic HBV infection [10]. The three most common types of kidney disease resulting from HBV infection are Membranous nephropathy, Membranoproliferative glomerulonephritis (MPGN) and Polyarteritis nodosa (PAN) [11]. Histologic patterns including MN, membranoproliferative glomerulonephritis (MPGN), immunoglobulin (Ig) A nephropathy, and focal segmental glomerulosclerosis (FSGS) have been reported [12, 13]. HBV‐associated renal disease most commonly manifests as MN, specifically HBV‐membranous nephropathy (HBV‐MN), which is linked to subepithelial deposits of viral immune complexes [14]. Differentiating it from primary MN can be aided by immunoglobulin G subclass analysis, as IgG4 typically dominates in primary MN but is non‐dominant in secondary forms, and by the identification of HBeAg and HBcAg within the glomerular deposits [15, 16]. While AA amyloidosis is an uncommon sequelae of chronic viral infections like hepatitis B, case reports have documented its occurrence [17, 18]. Histologically, AA amyloidosis is identified by congophilic deposits exhibiting green birefringence under polarized light and positive immunohistochemical staining for serum amyloid A (SAA) [19].

The coexistence of PLA2R‐positive MN and AA amyloidosis in a single patient with chronic HBV infection is exceptionally rare and presents profound diagnostic and therapeutic challenges. But reports of concurrent presence of renal amyloidosis with MN have been documented in literature [20]. The integration of clinical, serological, histopathological, and advanced immunohistochemical findings is critical for an accurate diagnosis in such complex cases of dual pathology. From a therapeutic standpoint, management must address both conditions: controlling the underlying HBV infection with antiviral agents (e.g., tenofovir, entecavir) while cautiously managing the immune‐mediated glomerulonephritis. The use of immunosuppressive agents like rituximab in HBV‐associated PLA2R‐positive MN has been explored and can be safe and effective, provided viral replication is suppressed and antiviral therapy is maintained to prevent reactivation [21]. However, the optimal treatment strategy for the rare confluence of MN and AA amyloidosis remains poorly defined due to its scarcity.

This case report describes a patient with the rare dual pathology of PLA2R‐positive MN and AA amyloidosis secondary to chronic HBV infection. By integrating detailed clinical, histopathological, and immunohistochemical findings with contemporary literature, this report aims to highlight the diagnostic complexity and therapeutic considerations required to navigate this challenging clinical scenario and achieve favorable outcomes.

## 2 Case History/Examination

A 38‐year‐old man presented to our hospital with a six‐month history of progressive generalized edema that initially involved the face and subsequently evolved into anasarca. His symptoms had fluctuated over the preceding year, with intermittent improvement occurring either spontaneously or with diuretic use. His history was notable for foamy urine, easy fatigability, and loss of appetite. He had been diagnosed with hepatitis B virus (HBV) infection 3 months prior and had initiated treatment with tenofovir alafenamide. A comprehensive review of systems was negative for joint pains, rash, fever, respiratory or gastrointestinal symptoms, jaundice, discolored urine, headache, chest pain, or syncope. He had no significant past medical history of tuberculosis, diabetes, or hypertension.

## 3 Differential Diagnosis, Investigation, and Treatment

Initial laboratory investigations performed at an outside facility revealed nephrotic‐range proteinuria (7980 mg/24 h), severe hypoalbuminemia (1.6 g/dL), and impaired renal function (serum creatinine 1.4 mg/dL). Serological evaluation confirmed active HBV infection (HBsAg‐positive, HBeAg non‐reactive, HBV DNA 4700 IU/mL). Additional findings included dyslipidemia (total cholesterol 255 mg/dL, LDL 165 mg/dL), an elevated erythrocyte sedimentation rate (ESR) of 35 mm/h, and a C‐reactive protein (CRP) level of 6.5 mg/L. Serologies for HCV, HIV, and VDRL were negative, and stool occult blood testing was unremarkable (Table 1). An echocardiogram and chest x‐ray were within normal limits. The patient was initially managed with tenofovir alafenamide and supportive care, including enalapril, atorvastatin, warfarin, and diuretics. Despite 4 months of this regimen, his condition paradoxically deteriorated. At the time of referral to our hospital, his serum creatinine had risen to 1.3 mg/dL, 24‐h urine protein had increased to 8835 mg, and serum albumin had declined to 0.8 g/dL.

**TABLE 1:** Longitudinal Clinical, Serological, and Histological Profile of a Patient with HBV, PLA2R Positive Membranous Nephropathy, and AA Amyloidosis.

Date	17/02/24	28/03/24	17/06/24	17/7/2024	24/10/24	23/06/25	11/07/25	5/08/25
24 h urine protein	7980	8835	4040	9207	9424	6120	6336	4304
Serum albumin	1.6	0.8	3.5	2.8	2.6	2.8	3.0	3.0
HBV DNA in IU/ML	4700			< 15			< 15	
Creatinine	1.3	1.4	0.6	0.9	1.0	1.0	0.9	1.0
Serum PLA2R					POS	NEG		
Treatment			Rituximab given (2 dose) 24/4/2024‐first dose 10/5/2024‐s dose		Cyclophosphamide+ Prednisolone‐started on 22/12/24			
	Kidney biopsy on 20/4/2024				Kidney biopsy on 21/10/2025			
Direct Immuno Fluorescence	IgA: Negative				IgA: Negative			
	IgG: 3+ capillary wall granular				IgG: 3+ capillary wall granular			
	Ig sub Class G1: 3+ capillary wall granular				Ig sub Class G1: 3+ capillary wall granular			
	Ig sub Class G2: Negative				Ig sub Class G2: Negative			
	Ig sub Class G3: Negative				Ig sub Class G3: Negative			
	Ig sub Class G4: 3+ capillary wall granular				Ig sub Class G4: 1+ capillary wall granular			
	IgM: Negative				IgM: Negative			
	C3: 1+ capillary wall granular				C3: 1+ capillary wall granular			
	C1q: Negative				C1q: Negative			
	Kappa light chains: 3+ capillary wall granular				Kappa light chains: 3+ capillary wall granular			
	Lambda light chains: 3+ capillary wall granular				Lambda light chains: 3+ capillary wall granular			
Immunohistochemistry	Staining for PLA2R (phospholipase A2 Receptor show diffuse granular positivity along glomerular capillary walls)				Staining for PLA2R (phospholipase A2 Receptor is negative along glomerular capillary walls)			
	Staining for SAA (serum amyloid associated) protein shows intense (3+) positivity along glomerular and extraglomerular sites of amyloid deposition				Smudgy entrapment of IGM is noted in few mesangial areas (corresponding to the amyloid deposits)			

**FIGURE 1:**
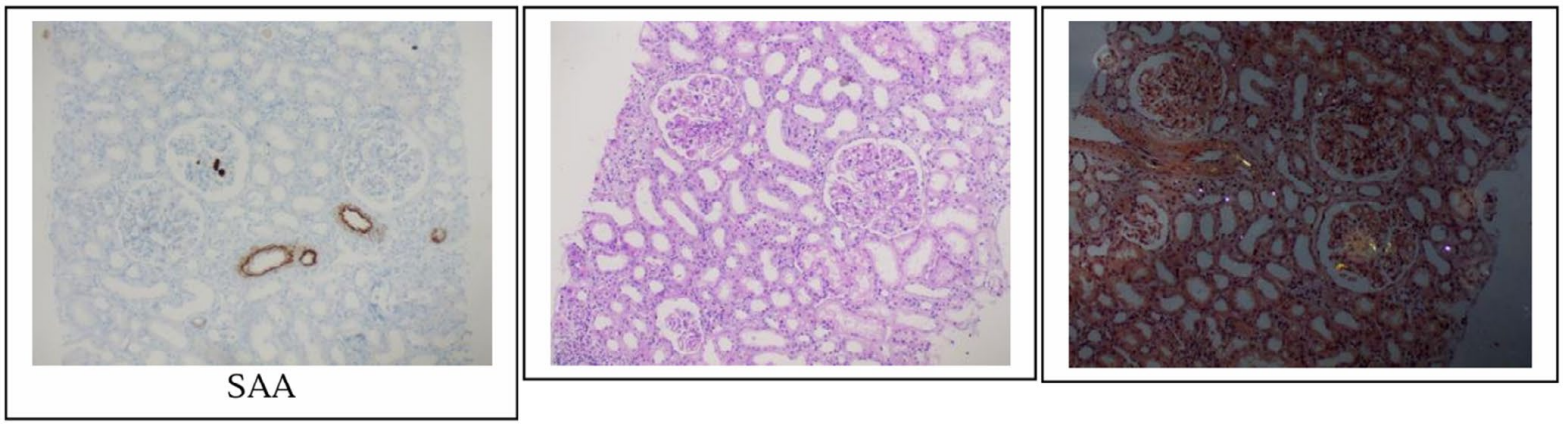
Histopathological findings demonstrating dual renal pathology. The left panel (Serum Amyloid A (SAA) immunohistochemistry (400×)) shows strong positivity within the glomerular mesangium and capillary loops, confirming the presence of AA‐type amyloid deposits. The middle panel (Periodic Acid‐Schiff (PAS) stain (400×)) highlights diffuse thickening of the glomerular basement membranes (GBM) and mesangial expansion, characteristic of a background membranous glomerulopathy pattern. The right panel (Congo red stain under polarized light (200×)) exhibits the pathognomonic “apple‐green” birefringence. This confirms the amyloid nature of the deposits.

Upon arrival at our hospital, a kidney biopsy was performed to establish a definitive diagnosis. Light microscopy of up to 18 glomeruli demonstrated diffuse capillary wall thickening with intramembranous mottling and mesangial expansion due to a pale eosinophilic material that was Congo red‐positive with apple‐green birefringence, diagnostic of amyloid deposition. Two glomeruli (11%) showed segmental tuft sclerosis, with no evidence of hypercellularity, thrombi, or crescents. Tubulointerstitial fibrosis involved 10%–15% of the cortex. Direct immunofluorescence revealed strong (3+) granular capillary wall staining for IgG, IgG1, IgG4, kappa and lambda light chains. It was negative for C1q and only showed 1+ granular pattern for C3. Immunohistochemistry was positive for phospholipase A2 receptor (PLA2R) along capillary walls and intensely positive (3+) for SAA protein in glomerular and vascular deposits, concluding a diagnosis of PLA2R‐positive membranous nephropathy with concurrent AA amyloidosis (Figure 1), (Table 1).

Following the biopsy results, the patient received two 1‐g doses of rituximab. Two weeks later, he demonstrated a significant clinical improvement, with proteinuria declining to 4040 mg/24 h and serum creatinine decreasing to 0.6 mg/dL, all while maintaining an undetectable HBV viral load. However, on subsequent follow‐up, his proteinuria increased again. Six months after rituximab therapy, a qualitative serum anti‐PLA2R antibody test returned positive. Due to the patient's financial constraints, a transition to an alternative, hospital‐available immunomodulatory regimen was necessary. He was started on cyclophosphamide and prednisolone for a six‐month course. While this treatment improved his edema, significant proteinuria persisted at 6336 mg/24 h, with a stable serum creatinine of 1.0 mg/dL. Of note, repeat serological testing at this juncture demonstrated that serum anti‐PLA2R antibodies had become negative. Immunosuppressive therapy was subsequently discontinued, and he was maintained on maximum doses of antiproteinuric agents, including an ACE inhibitor, dapagliflozin, and finerenone.

**FIGURE 2:**
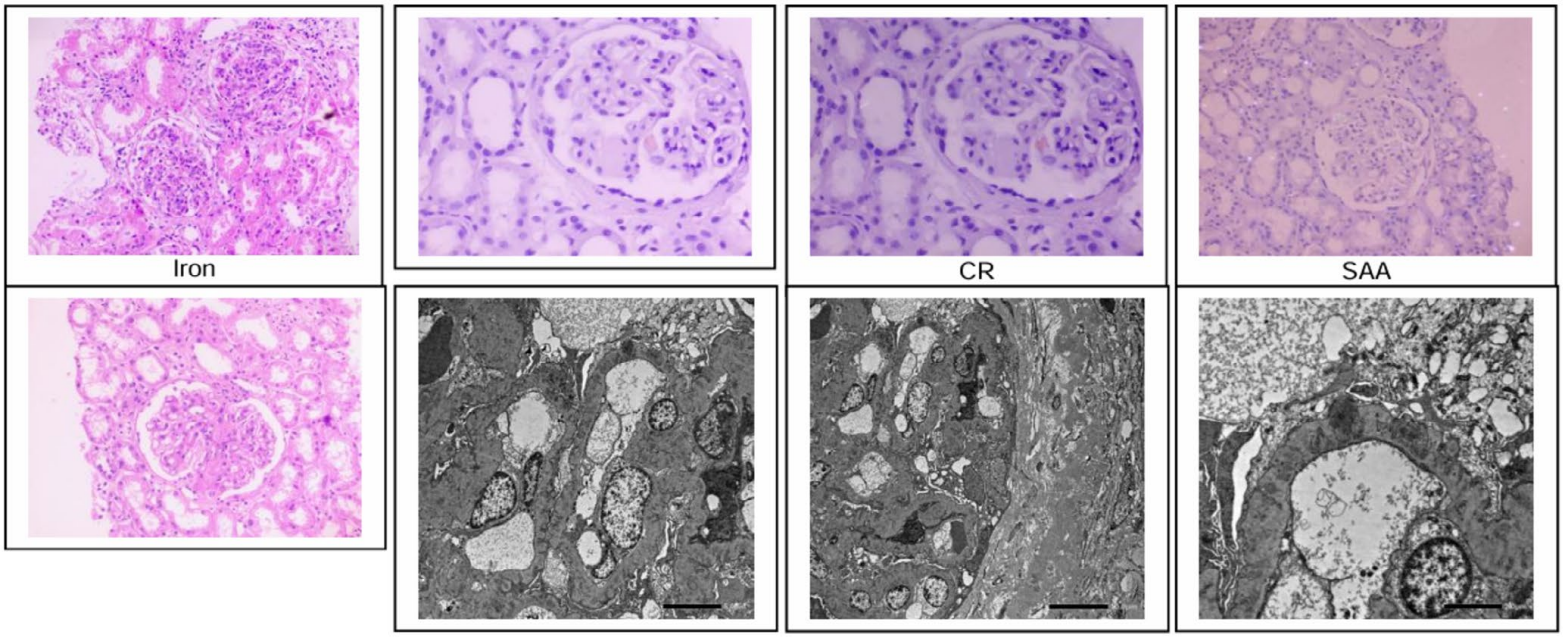
Histological and ultrastructural confirmation of dual renal pathology. Top row (left to right): The first panel (Hematoxylin and Eosin (H&E) staining) shows glomeruli with mesangial expansion and amorphous, eosinophilic extracellular deposits. The second panel (Periodic Acid‐Schiff (PAS) stain) highlights diffuse thickening of the glomerular basement membranes. The third panel (Congo red staining) confirms the presence of amyloid deposits. The fourth panel (Serum Amyloid A (SAA) immunohistochemistry) demonstrates strong positivity, identifying the deposits as AA‐type amyloid. Bottom row (left to right): The first panel (Hematoxylin and Eosin (H&E) staining) shows glomeruli with similar features to the top‐left panel. The second panel (Low‐power electron micrograph) reveals extensive subepithelial electron‐dense deposits and global podocyte foot process effacement. Extraglomerular electron‐dense deposits are not seen. The third panel (High‐power magnification) confirms finely granular and amorphous in nature and with no definite substructure. The fourth panel (Ultrastructural analysis) shows focal microvillous change of terminal process of visceral epithelial cells. Endothelial cells do not showtubloreticular inclusions.

A second kidney biopsy was subsequently performed to investigate the discordance between immunological remission (negative serum PLA2R) and persistent heavy proteinuria. Light microscopy again revealed a membranous pattern glomerulopathy, but with an increased proportion of segmentally sclerosed glomeruli (33.3%). Amyloid deposits were noted to be scant and focal. Immunofluorescence findings remained similar to the first biopsy. Interestingly, immunohistochemistry was now negative for PLA2R, THSD7A, NELL‐1, and Sema 3b. Electron microscopy confirmed features consistent with stage 2 membranous nephropathy. The overall impression, comparing both biopsies, was of a marked reduction in amyloid burden and the complete disappearance of PLA2R antigen, indicating immunological remission of the membranous nephropathy. The predominant pathology now appears to be resolving AA amyloidosis with secondary sclerotic injury. The patient is currently managed with maximum supportive care (Figure 2).

## 4 Conclusion and Results

This complex case of a patient with concurrent Hepatitis B virus infection, PLA2R‐positive MN, and AA amyloidosis provides critical insights into the diagnostic and therapeutic challenges of dual glomerular pathology. The initial presentation with overlapping immunopathological features, specifically the co‐presence of IgG1, IgG4, and PLA2R, created a diagnostic dilemma between a secondary HBV‐associated MN and a primary MN with coincidental HBV. The definitive lack of proteinuria response to antiviral therapy alone, followed by a significant clinical and immunological remission only after B‐cell depletion with rituximab (evidenced by the disappearance of circulating anti‐PLA2R and glomerular PLA2R antigen), strongly supports the diagnosis of a primary, autoantibody‐driven MN. Furthermore, the concomitant reduction in amyloid burden underscores that effective suppression of the underlying autoimmune and inflammatory drive is paramount, beneficially impacting both disease processes. This case highlights that in such scenarios, a tailored, sequential therapeutic strategy, prioritizing antiviral control to prevent reactivation while employing immunosuppression based on immunologic response, is essential, and that persistent proteinuria likely reflects irreversible structural damage rather than ongoing immunologic activity.

## 5 Discussion

The primary objective of this case report is to delineate the diagnostic and therapeutic challenges posed by the rare coexistence of PLA2R positive primary MN and AA amyloidosis in a patient with chronic HBV infection. It highlights the necessity of distinguishing between virus associated secondary glomerulopathy and coincidental primary autoimmune disease, particularly in regions where HBV is endemic. The rare convergence of these three distinct renal pathologies challenges the conventional approach that favors a single unifying etiology. By documenting the patient's clinical and histological evolution with repeat biopsy, this report provides a framework for managing overlapping glomerulopathies using sequential antiviral and immunosuppressive therapy.

Primary MN is an autoantibody‐mediated disease, with antibodies targeting the M‐type PLA2R detectable in 70%–80% of patients and serving as a highly specific marker for the primary form of the disease [3, 7, 22]. However, HBV infection is a well‐established secondary cause of MN in endemic regions, and some studies report positive PLA2R in HBV‐associated MN [15, 23]. Our patient's presentation with both circulating anti‐PLA2R antibodies and positive glomerular PLA2R staining aligns with emerging evidence suggesting a pathogenic overlap between primary and HBV‐associated MN [8, 15]. The granular PLA2R staining observed, sometimes co‐localized with HBsAg, further supports a potential interaction between viral antigens and the podocyte target [8]. Although HBV antigen deposits (HBsAg, HBcAg, HBeAg) were not investigated on the renal biopsy, the presence of active HBV infection (positive HBsAg and/or detectable HBV DNA) supports a secondary etiology.

The immunopathological profile in our patient presented a complex diagnostic intersection between classic secondary features and primary pattern markers. On one hand, the granular distribution of IgG1 and C3 along the glomerular capillary loops provides some support for a secondary MN linked to chronic hepatitis B [8]. On the other hand, this must be weighed against the PLA2R positivity and IgG4 predominance, which are highly specific for primary MN. A critical diagnostic pivot was the absence of C1q deposition: in classic HBV‐associated secondary MN, immunopathology typically demonstrates a predominance of IgG1 and robust C1q deposition, reflecting classical complement pathway activation alongside weaker IgG4 staining [16, 24, 25]. In contrast, the simultaneous presence of IgG1, IgG4, and PLA2R in our patient, coupled with C1q negativity, suggests an overlapping immunobiological feature. This hybrid profile likely reflects one of three possibilities: a coincidental overlap of primary MN with chronic HBV; an early transitional stage of primary MN characterized by an incomplete IgG subclass switch from IgG1 to IgG4; or a rare variant of secondary MN presenting with atypical immunopathology [24, 25, 26, 27]. Ultimately, the dominance of the PLA2R/IgG4 axis led us to treat this as a primary‐pattern MN, a distinction that fundamentally dictated therapeutic escalation to B‐cell depletion therapy.

Given the diagnostic uncertainty between primary and secondary MN, the therapeutic response to antiviral therapy served as a clinically useful discriminator. Multiple clinical studies have demonstrated proteinuria remission rates exceeding 60% with antiviral therapy, as well as a strong correlation between viral suppression and improvement of the nephrotic syndrome [28, 29, 30]. More specifically, in HBV‐associated membranous nephropathy, significant proteinuria reduction typically begins as early as 12 weeks, with remission rates of more than 50% by 24 weeks [31, 32, 33]. Our patient showed no clinical improvement after 16 weeks of tenofovir therapy, arguing strongly against secondary HBV‐MN and supporting coincidental primary MN. Furthermore, the disappearance of PLA2R from both serum and renal tissue after immunosuppressive therapy is consistent with immunological remission of primary MN [24, 34]. Thus, in this clinical context, the lack of proteinuria response to antivirals, combined with PLA2R/IgG4 positivity and subsequent post‐immunosuppression PLA2R clearance, strongly supports primary MN with coincidental chronic HBV infection rather than secondary HBV‐associated MN.

The reduction in glomerular IgG4 staining from 3+ to 1+ after immunomodulatory treatment was a clinically significant finding, indicating a favorable immunologic response. IgG4 is the predominant pathogenic autoantibody subclass in PLA2R‐associated disease, and its intra‐glomerular presence directly correlates with disease activity and severity. Thus, a decrease in IgG4 reflects a reduced pathogenic autoantibody burden, a change strongly associated with higher rates of clinical remission and improved renal outcomes [35, 36, 37]. This histologic observation aligns with quantitative studies demonstrating that a decline in circulating anti‐PLA2R IgG4 levels and the IgG4‐to‐IgG ratio are superior predictors of remission compared to total anti‐PLA2R IgG measurements alone [33, 34]. Consequently, achieving low or negative IgG4 after treatment marks therapeutic efficacy and likely diminishes complement activation via the lectin pathway, a key mechanism of podocyte injury [35, 37, 38].

However, the persistence of granular IgG1 at 3+ intensity on repeat biopsy, despite IgG4 decline and PLA2R disappearance, is noteworthy. IgG1 is typically more prominent in early or atypical stages of MN, and its persistence after therapy may indicate residual immune activity, epitope spreading, or a shift in autoantibody profile [39, 40]. IgG1 deposits are potent activators of the classical and alternative complement pathways, which may contribute to ongoing glomerular injury even as the dominant IgG4 response declines [39]. Some studies suggest that patients with persistent IgG1 or IgG3 deposits have less favorable responses to standard immunosuppression and may need more intensive or prolonged regimens [40]. Several interpretations are possible: true residual autoimmunity against an unidentified podocyte antigen; epitope spreading, which is associated with refractory disease and poorer prognosis [41, 42]; or burnt‐out non‐pathogenic deposits that persist despite clearance of the inciting antigen. Nevertheless, in our patient, where the target antigen has cleared and circulating autoantibodies are undetectable, the persistent IgG1 deposits are best interpreted as residual non‐pathogenic staining rather than evidence of ongoing active disease.

The co‐existence of renal AA amyloidosis in our patient adds another unusual and complex dimension. AA amyloidosis arises from the chronic inflammatory state driven by sustained elevations in serum SAA protein [43]. Although typically associated with chronic inflammatory diseases, reports of amyloidosis with chronic HBV infection exist [17, 18]. In our patient, the absence of any other chronic inflammatory condition implicates HBV as the driver of AA amyloidosis, an association infrequently documented. Diagnosis was confirmed histologically by Congo red positivity with apple‐green birefringence and intense SAA immunohistochemistry. Treatment of AA amyloidosis centers on controlling the underlying inflammation; antiviral therapy to suppress HBV replication is the primary intervention when chronic hepatitis B is the etiology [17, 44]. This aligns with our patient's clinical response, in whom amyloid deposition was significantly reduced after antiviral therapy.

A limitation of this study is the absence of direct immunohistochemical or immunofluorescence staining for HBV antigens (HBsAg, HBcAg, HBeAg) on the kidney biopsy, which could have provided direct evidence of viral antigen deposition within glomeruli.

In conclusion, this case provides several instructive lessons for managing renal disease with overlapping pathologies. First, the coexistence of PLA2R‐positive MN and AA amyloidosis with chronic HBV highlights profound diagnostic complexity in endemic regions, where a viral infection may be a pathogenic driver, immune trigger, or coincidental finding. The initial immunologic pattern, absence of C1q and dominance of the IgG4/PLA2R axis, provided a critical diagnostic pivot toward a primary autoimmune process. Second, clearance of anti‐PLA2R antibodies and glomerular PLA2R antigen after immunomodulatory therapy confirmed the primary nature of the MN. However, reduction of the dominant IgG4 response may not equate to complete immunologic remission, as persistent IgG1 raises the possibility of ongoing, albeit altered, autoimmune activity. Crucially, the discordance between persistent IgG1, negative serum anti‐PLA2R, and resolved PLA2R antigen indicates that residual proteinuria is no longer driven by active autoimmune injury. Instead, heavy proteinuria is best explained by irreversible structural damage: a substantial increase in globally sclerosed glomeruli (from 11% to 33.3%) and permanent disruption of the filtration barrier by residual amyloid deposits and fibrosis. This interpretation underscores the critical role of repeat renal biopsy in documenting resolution of active membranous disease and evolution to predominantly sclerotic injury. Thus, an integrated approach combining longitudinal serology with detailed histological follow‐up, including IgG subclass analysis, is essential to distinguish active immunologic response from permanent structural damage and to avoid unnecessary immunosuppression in overlapping autoimmune and infectious renal diseases.

## Author Contributions

**Betelhem Abreham:** conceptualization, data curation, investigation, project administration, supervision, writing – original draft, writing – review and editing. **Leja Hamza:** conceptualization, supervision, validation, writing – review and editing. **Azeb Kebede:** conceptualization, supervision, validation, writing – review and editing. **Daniel Rebuma Bekele:** investigation, validation, writing – review and editing. **Zekarias Amdemariam:** conceptualization, investigation, project administration, validation, visualization, writing – review and editing.

## Funding

The authors have nothing to report.

## Ethics Statement

Our institution does not require ethical approval for reporting individual cases or case series.

## Consent

Written informed consent was obtained from the patient to publish this report in accordance with the journal's patient consent policy.

## Conflicts of Interest

The authors declare no conflicts of interest.

## Data Availability

The data that support the findings of this case report are available from the corresponding author upon reasonable request.
